# Draft genome sequence of a human isolate of *Taiozyma onychis*

**DOI:** 10.1128/mra.00767-26

**Published:** 2026-08-14

**Authors:** Steve A. James, David J. Baker, Chloe Uyl, Simon R. Carding, Alessia Buscaino

**Affiliations:** 1 Food, Microbiome and Health Programme, Quadram Institute Bioscience 7308, Norwich, United Kingdom; 2 Centre for Microbial Interactions, Quadram Institute Bioscience 7308, Norwich, United Kingdom; 3 Norwich Medical School, University of East Anglia 6106, Norwich, United Kingdom; Rochester Institute of Technology, Rochester, New York, USA

**Keywords:** *Taiozyma onychis*, fungi, gut mycobiota, gut commensal, opportunistic pathogen

## Abstract

*Taiozyma onychis* (syn. *Wickerhamomyces onychis*) is a rare opportunistic human fungal pathogen. Here, we report the draft genome sequence of *T. onychis* NCYC 4433, a fecal isolate from a 62-year-old female.

## ANNOUNCEMENT

*Taiozyma onychis* (1), formerly *Wickerhamomyces onychis* (2), is an ascomycetous yeast that has been isolated from both the environment and humans (2–4). Found in the human gut (3), it can also be an opportunistic pathogen, albeit a rare one (2, 4). Here, we combined short- and long-read sequencing to obtain the genome sequence of *T. onychis* NCYC 4433, a fecal isolate from a 62-year-old female participant of the MOTION Study (5). A fecal homogenate was prepared in sterile phosphate-buffered saline (PBS) and cultured on Sabouraud dextrose (SD) agar plates containing penicillin (25 U/mL) and streptomycin (25 U/mL) and incubated at 30°C. Species identity from single colonies (with identical morphotype) was determined by PCR amplification and Sanger sequencing of the ribosomal DNA internal transcribed spacer (ITS) region (accession number OZ481044.1) using primers ITS1F and ITS4 (6, 7). Sequence identity was 99.45% (542 nt overlap) to the *T. onychis* type strain NRRL Y-7123^T^ (KY105910).

Genomic DNA was isolated using a MasterPure Yeast DNA Purification Kit (Cambio) following the manufacturer’s instructions with additional lyticase (100 U) and proteinase K treatment steps from 1.2 mL of a 2-day liquid SD culture grown at 30°C (at 180 rpm). DNA concentration and purity were assessed using a Qubit 4.0 fluorometer (Thermo Fisher Scientific) and a NanoDrop 1000 spectrophotometer (Thermo Fisher Scientific). A short-read library was prepared using a NextSeq 1000/2000 P2 XLEAP-SBS Reagents Kit (300 cycles) (Illumina), with DNA normalized to 5 ng/μL using a 20-fold diluted Illumina DNA Prep Kit protocol (8), barcoded using 10 bp unique dual indexes (UDIs), and sequenced on a NextSeq 2000 instrument. A Nanopore library was prepared from the same gDNA prep without fragmentation or size selection using the SQK-LSK109 1D Ligation Kit (Oxford Nanopore Technologies, ONT) and sequenced on a PromethION instrument (ONT) with a FLO-PRO114M flow cell (ONT). Base calling was performed using Guppy (ONT; v3.6.0) in high-accuracy mode (model dna_r9.4.1_450bps_hac).

**Fig 1:**
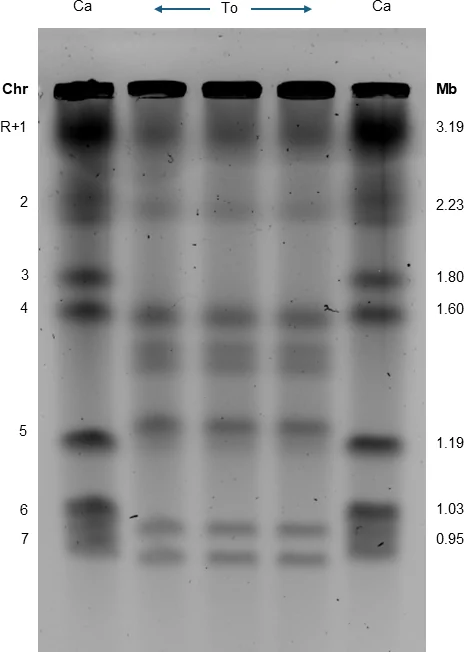
Karyotype of *T. onychis* NCYC 4433 (To; lanes 2–4) and *C. albicans* SC5314 (Ca; lanes 1 and 5; size standard) on an ethidium bromide-stained gel (inverted image). *C. albicans* chromosome numbers are shown on the left side, while chromosome sizes (in megabases) are shown on the right side. For each strain, yeast cells were embedded in low-melt agarose, and then digested away to intact chromosome-sized DNA that was separated by clamp homogeneous electric field electrophoresis (15).

Default parameters were used unless otherwise noted. Illumina reads were filtered with fastp v.0.23.2 (9). Nanopore reads were filtered using NanoFilt v.2.3.0 (10), selecting reads with quality ≥10 and length ≥20,000 bp. *De novo* assembly of the filtered Nanopore reads was performed using Flye v2.9.1 (11) using long reads in ‘ONT regular reads, pre-Guppy5’ mode. The assembly was polished with the Illumina data using three rounds of alignment with BWA-MEM v.0.7.17.1 (12) and Pilon v.1.24 (13) correction. Key sequencing and assembly metrics are shown in Table 1. The assembly includes 10 contigs with a putative telomere repeat sequence (5′- GACACCACACACATCCA-3′)_n_ identified using Tandem Repeats Finder v.4.09 (14) at one end. Genome completeness was estimated at 95.0% using BUSCO v.5.5.0 (15) with the saccharomycete_odb10 data set. Electrophoretic karyotyping indicates this strain has eight to nine chromosomes similar in size range to *Candida albicans* SC5314 (Fig. 1) (16).

**TABLE 1:** Summary of key sequencing and assembly outputs

Metric	Value
Illumina	
Total number of reads	9,679,076
N50 (no. of bases)	150
Total bases (Gb)	1.45673
Genome coverage^*a*^	118.4
Oxford Nanopore	
Total number of reads	1,059,522
Number of filtered reads^*b*^	134,025
N50 (no. of bases)	29,547
Total bases (Gb)	3.95475
Genome coverage^*a*^	321.5
Assembled genome	
Number of contigs	27
Total genome size (no. of bases)	12,466,078
N50 (no. of bases)	860,330
G + C content (%)	41.15

^*a*^ Based on the 12.3 Mb genome of *T. onychis* NRRL Y-7123^T^ (GCA_030582435.1).

^*b*^ Reads with quality ≥10 and length ≥20,000 bp (filtered using NanoFilt v.2.3.0).

The NCYC 4433 assembly (GCA_984806805.1) has an average nucleotide identity (17) of 97.66% to the NRRL Y-7123^T^ assembly (GCA_030582435.1) (18). Augustus v.3.2.3 (19) predicted 5,672 protein-coding genes using the *Scheffersomyces stipitis* training data set, and 189 tRNA genes were detected using tRNAscan SE 2.0 (20).

## Data Availability

This whole-genome shotgun project has been deposited in DDJB/ENA/GenBank (BioProject number PRJEB114437, assembly accession number CFJMSG010000000). The version described in this paper is version 1. The raw reads are available in the NCBI Sequence Read Archive (SRA) under accession numbers ERR17369218 (ONT) and ERR17369219 (Illumina). The predicted protein and tRNA annotation data sets have been deposited in Zenodo (https://doi.org/10.5281/zenodo.21472325).
